# Alteration in Photosynthetic and Yield Parameters, Content of Metabolites, and Antioxidant Activity of Pepper (*Capsicum annuum*): Effect of Bio-Organic Substrate and Depolymerized Chitosan

**DOI:** 10.3390/molecules30244768

**Published:** 2025-12-13

**Authors:** Piotr Salachna, Agnieszka Zawadzińska, Rafał Piechocki, Małgorzata Mikiciuk, Julita Rabiza-Świder, Ewa Skutnik, Łukasz Łopusiewicz

**Affiliations:** 1 Department of Horticulture, West Pomeranian University of Technology in Szczecin, Słowackiego 17, 71-434 Szczecin, Poland; agnieszka.zawadzinska@zut.edu.pl; 2 Faculty of Environmental Management and Agriculture, West Pomeranian University of Technology in Szczecin, Słowackiego 17, 71-434 Szczecin, Poland; rafal.piechocki@zut.edu.pl; 3 Department of Bioengineering, West Pomeranian University of Technology in Szczecin, Słowackiego 17, 71-434 Szczecin, Poland; malgorzata.mikiciuk@zut.edu.pl; 4 Section of Ornamental Plants, Institute of Horticultural Sciences, Warsaw University of Life Sciences, Nowoursynowska 166, 02-787 Warsaw, Poland; julita_rabiza_swider@sggw.edu.pl (J.R.-Ś.); ewa_skutnik@sggw.edu.pl (E.S.); 5 School of Medical & Health Sciences, Vizja University, Okopowa 59, 01-043 Warsaw, Poland; l.lopusiewicz@vizja.pl; 6 Department Pharmaceutical Biology, Institute of Pharmacy, University of Greifswald, Friedrich-Ludwig-Jahn-Str. 17, 17489 Greifswald, Germany

**Keywords:** bell pepper, phytochemicals, organic substrate, chitooligosaccharides, biostimulant, elicitor

## Abstract

Peppers are of substantial economic importance and hold a prominent position among vegetables rich in health-promoting compounds, which drives continuous efforts to develop improved cultivation strategies. The study aimed to determine the effects of substrate type and depolymerized chitosan on the physiological parameters, the chemical composition of leaves and fruits, and the yield of two bell pepper cultivars: ‘Marta Polka’ and ‘Oda’. The plants were grown in a 100% peat substrate and in a mixture of peat, wood fiber (*Pinus sylvestris*), and green compost (2:1:1 *v*/*v*/*v*), with or without drenching with a solution of depolymerized chitosan. Results indicated that the growing medium, chitosan application, cultivar type, and their interactions altered several physiological, morphological, and biochemical traits. The highest total fruit weight fresh (471.23 g plant^−1^) was obtained for the ‘Marta Polka’ cultivar grown in peat drenched with chitosan, whereas the lowest (192.02 g plant^−1^) was recorded for ‘Oda’ grown in a substrate mix without the biostimulant. Net CO_2_ assimilation rate, stomatal conductance, fresh weight of fruit, and antioxidant activity (ABTS and FRAP assays) were improved in the ‘Oda’ cultivar grown in the substrate mix and treated with depolymerized chitosan compared with plants grown in 100% peat without chitosan. The ‘Marta Polka’ plants grown in the substrate mix and treated with chitosan had a higher net CO_2_ assimilation rate, photosynthetic water-use efficiency, total free amino acid content, and antioxidant activity (FRAP assay) than those grown in peat alone and not treated with the biostimulant. The results demonstrate that both substrate composition and the response to depolymerized chitosan are cultivar-specific, and that wood fiber and compost can serve as ecological alternatives to peat, enhancing overall pepper fruit quality.

## 1. Introduction

Biostimulants are diverse substances that stimulate plant growth, increase their resistance to diseases and stresses, and have a beneficial effect on crop size and biological quality [1,2]. In plant production, especially sustainable production, the use of biostimulants is considered an environmentally friendly agricultural practice [3,4]. The biostimulant market is estimated to reach USD 8.02 billion in 2030 [5]. The primary driver of biostimulant market growth is the expanding organic food sector [4]. There is a growing worldwide emphasis on the importance of healthy lifestyles and the benefits of healthy eating. In addition, the expected growth in the world’s population to over 8.5 billion by 2030 underscores the importance of food security, high productivity, and increased crop yields [5]. Despite its growing popularity, the biostimulant market faces serious regulatory challenges [6]. The absence of uniform legal regulations allows products of unknown origin and with undefined active-substance composition to enter the market [5,6]. It can lead to unpredictable effects, which, in turn, call into question the safety and effectiveness of these preparations [7]. The use of biostimulants with predictable, repeatable effects is crucial for producers’ confidence and the further development of this market segment [5,6,8].

Chitosan is a biostimulant that is safe for the environment and humans [9]. Chitosan can have a positive effect on many physiological and metabolic processes in plants, increasing growth, yield, photosynthetic intensity, photosynthetic pigment content, nutrient levels, and secondary metabolite production [10,11,12,13,14]. Chitosan is believed to play an important role in increasing plant resistance to adverse biotic and abiotic factors [15,16,17]. A limitation in the use of chitosan as a biostimulant is its very poor solubility in water [18,19]. Chitosan is most often dissolved in acetic acid and applied as a solution, and its effect on plants may result from the acid rather than the biostimulant. As is well known, acetic acid applied exogenously in low concentrations (10 mM–100 mM) increases plant resistance to abiotic stresses and acts as a biostimulant [20]. There are significantly more studies on the reaction of plants to chitosan dissolved in acetic acid than studies focusing on the assessment of the effect of pure chitosan on plant growth, physiology, and metabolism [21,22,23]. Another issue is that many studies on the use of chitosan in plant cultivation lack essential information on the physicochemical properties of the chitosan used.

From a chemical point of view, chitosan is a polymer obtained by deacetylation of the homopolysaccharide chitin, via chemical, biological, or enzymatic processes. These processes lead to the hydrolysis of acetylamine groups NH–C(=O)–CH_3_ with the formation of functional amino groups -NH_2_ [24]. Chitosan is considered a polymer that, upon deacetylation, contains less than 50% N-acetylglucosamine residues [25]. The physicochemical properties of chitosan depend on the size of the macromolecules and their distribution [26]. The higher the molecular weight, the more chitosan tends to form inter- and intramolecular hydrogen bonds between sugar chains, leading to a decrease in its solubility in water. A reduction in molecular weight below 30 kDa results in chitosan that is easily soluble in water [24,25,26]. According to current research, water-soluble chitosan with a low molecular weight can stimulate plant growth and development, increase yield, affect physiological processes and metabolism, intensify the biosynthesis of specialized metabolites, and activate immune mechanisms, with the effect of the biostimulant depending on its physicochemical properties, genotype, dose, method of application, and growing conditions [27,28,29,30,31].

Bell peppers (*Capsicum annuum* L.) are gaining increasing consumer interest due to their desirable flavor, high nutritional value, and health-promoting properties [32]. They are also cultivated as ornamental plants due to the diverse colors, shapes, and sizes of their fruits. Pepper fruits are a valuable source of natural antioxidants, carotenoids, flavonoids, polyphenols, vitamins, and macro- and microelements, the content of which depends on the cultivar, fruit color, fertilization, substrate, and cultivation methods [33,34,35]. Peppers are grown on a commercial scale in fields and under cover, as well as in home gardens, community gardens, and urban farms. There is demand for solutions that enable peppers to be produced in the most sustainable, environmentally friendly way [36,37]. An environmentally friendly method of improving pepper growth and yield is the use of biostimulants [38], including chitosan [39,40,41], oligochitosan [42,43,44], and their derivatives [45,46,47].

Environmental considerations and the rising cost of peat as a growing medium in horticulture have prompted the search for high-quality, inexpensive alternative substrates for pepper cultivation [37,48,49]. Wood fiber is a product of the wood industry, a renewable and environmentally friendly raw material, and its physical and chemical properties are similar to those of peat. For these reasons, wood fiber can constitute up to 50% of horticultural substrates [50]. In general, wood fiber is produced from pine, fir, or spruce due to their lower content of phytotoxic compounds compared to deciduous tree species [51,52]. Composts are also gaining popularity as a sustainable alternative to peat [53]. Although composts are characterized by unstable composition, they are considered promising additives to substrates due to their nutrient content. Many studies confirm the usefulness of composts in pepper cultivation [54,55,56,57]. However, there is limited information on the use of pine wood fiber as a substrate component in pepper production.

No information is available on the effects of water-soluble chitosan applied to the soil and on the type of substrate on the growth, yield, and quality of bell peppers. Therefore, the objective of this study was to evaluate how depolymerized chitosan drench application and the incorporation of wood fiber and green compost into commercial potting substrate affect bell pepper performance, including leaf photosynthetic gas exchange, fruit yield, and biochemical traits. Two bell pepper cultivars differing in fruit color were used in the experiment, namely ‘Marta Polka’ (orange) and ‘Oda’ (purple).

## 2. Results

### 2.1. Effect of Medium, Chitosan and Cultivar on Photosynthetic Parameters

**Figure 1:**
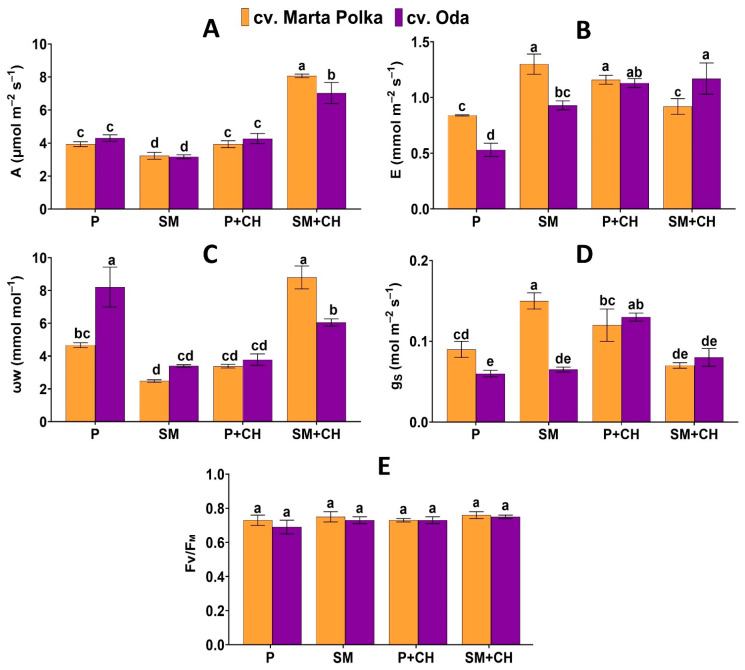
The interaction effects of media, chitosan and cultivar on (**A**) assimilation intensity CO_2_ net (**A**); (**B**) transpiration intensity (**E**); (**C**) photosynthetic water utilization factor (ω_W_), (**D**) stomatal conductivity H_2_O (g_s_), and (**E**) maximum photochemical efficiency (F_V_/F_M_) of pepper plants. Data are mean ± SD (n = 3). Different letters above the error bars indicate significant differences for *p* ≤ 0.05 (ANOVA and Tukey’s post hoc test). P = peat; SM = substrate mix; CH = chitosan.

Data presented in Figure 1, Table 1 clearly shows that assimilation intensity CO_2_ net, transpiration intensity, stomatal conductivity and photosynthetic water utilization factor were significantly affected by experimental factors and their interaction effects. The highest values of assimilation intensity CO_2_ net (8.07 μmol m^−2^ s^−1^) and photosynthetic water utilization factor (8.80 mmol mol^−1^) were recorded for the ‘Marta Polka’ cultivar treated with depolymerized chitosan, growing in a substrate mix composed of peat, wood fiber, and green compost. The highest transpiration intensity (1.30 mmol m^−2^ s^−1^) and stomatal conductivity (0.15 mol m^−2^ s^−1^) were observed in the ‘Marta Polka’ cultivar growing in a substrate mix and not treated with a biostimulant. What is worthwhile, both cultivars grown in the substrate mix and treated with depolymerized chitosan exhibited increased CO_2_ net assimilation compared with other treatments (Figure 1).

Irrespectively of the biostimulant and cultivars studied, plants growing in a substrate mix compared to plants growing in 100% peat, were characterized by significantly higher CO_2_ net assimilation and transpiration intensity. They also showed a slightly higher value of the chlorophyll fluorescence parameter (F_V_/F_M_) and, at the same time, reduced stomatal conductivity compared to plants growing in 100% peat (Table 1).

When assessing the main effect of depolymerized chitosan on the physiological indicators of pepper leaves, it was found that the use of the biostimulant caused a significant increase in CO_2_ net assimilation, transpiration, and the photosynthetic water utilization factor in plants compared to plants not treated with the biostimulant (Table 1).

Regardless of growing medium and chitosan applied, the leaves of the ‘Marta Polka’ cultivar had higher transpiration intensity and stomatal conductance as compared with the ‘Oda’ cultivar. In turn, plants of the ‘Oda’ cultivar had higher values of the photosynthetic water utilization factor than plants of the ‘Marta Polka’ cultivar (Table 1).

### 2.2. Effect of Medium, Chitosan and Cultivar on Photosynthetic Pigments, Reducing Sugars, Total Phenolic, and Total Free Amino Acid Contents in Pepper Leaves

**Figure 2:**
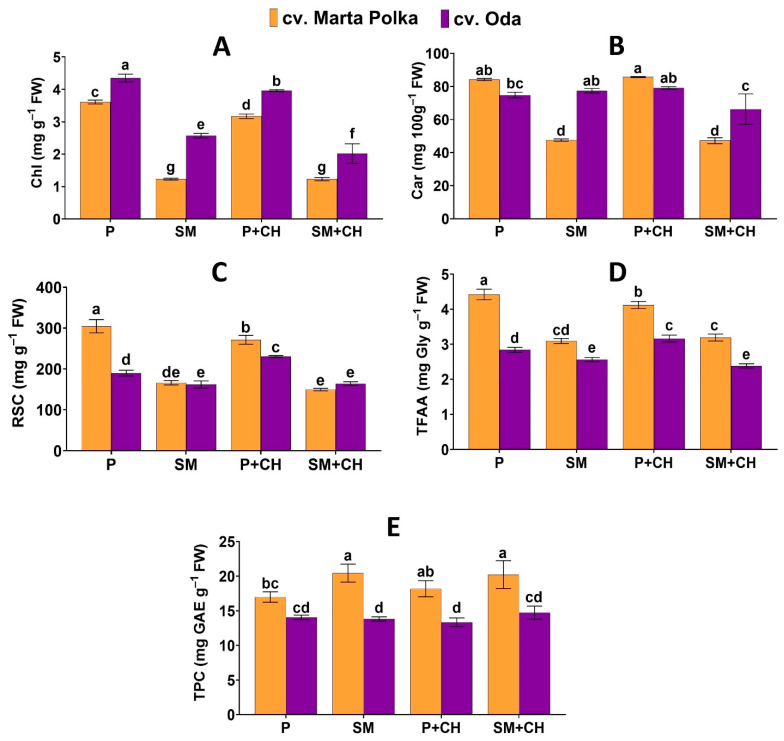
The interaction effects of media, chitosan and cultivar on (**A**) total chlorophyll content (Chl); (**B**) total carotenoids content (Car); (**C**) reducing sugars content (RSC), (**D**) total free amino acid content (TFAA), and (**E**) total phenolic content (TPC) in the leaves of pepper. Data are mean ± SD (n = 3). Different letters above the error bars indicate significant differences for *p* ≤ 0.05 (ANOVA and Tukey’s post hoc test); P = peat; SM = substrate mix; CH = chitosan; FW = fresh weight; Gly = glycine; GAE = gallic acid equivalents.

Results of treatments effects on the biochemical characteristics of the leaves are summarized in Figure 2 and Table 2. Based on the interaction, the highest chlorophyll content in the leaves (4.35 mg g^−1^ FW) was found in plants of the ‘Oda’ cultivar growing in 100% peat and not treated with a biostimulant. The highest carotenoid content in leaves was found in the ‘Marta Polka’ cultivar growing in 100% peat and treated with depolymerized chitosan (85.71 mg 100 g^−1^ FW). Among all treatments, the highest content of reducing sugars (304.57 mg g^−1^ FW) and total free amino acids (4.42 mg Gly g^−1^ FW) were found in the leaves of the ‘Marta Polka’ cultivar grown in pure peat and not drenched with a depolymerized chitosan solution. The highest total polyphenol content was found in the leaves of the ‘Marta Polka’ cultivar grown in substrate mix with chitosan (20.22 mg GAE g^−1^ FW) and without a biostimulant (20.44 mg GAE g^−1^ FW) (Figure 2).

An analysis of the main effect of the substrate used in the experiment showed that plants growing in 100% peat had increased content of chlorophyll, carotenoids, reducing sugars, and total free amino acids, while at the same time having a reduced total polyphenol content compared to plants grown in substrate mix (Table 2).

Grand means presented in Table 2 clearly show that the leaves of different pepper cultivars varied significantly in their chemical composition. The leaves of the ‘Oda’ cultivar had more chlorophyll and carotenoids compared to the leaves of the ‘Marta Polka’ cultivar. In turn, the leaves of the ‘Marta Polka’ cultivar contained more reducing sugar, total free amino acids, and total phenolic content than the leaves of the ‘Oda’ cultivar (Table 2).

### 2.3. Effect of Medium, Chitosan and Cultivar on Pepper Yield

**Figure 3:**
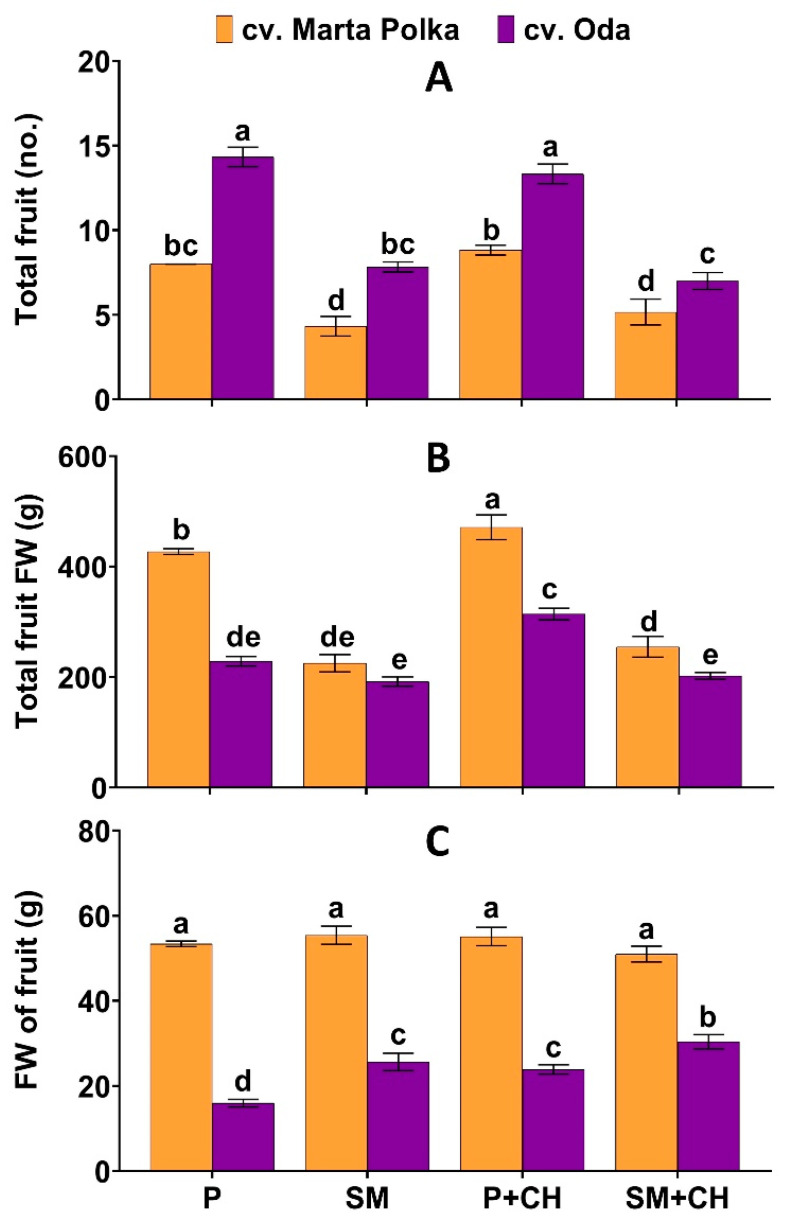
The interaction effects of media, chitosan and cultivar on (**A**) total fruit number per plant; (**B**) total fresh weight of fruits per plant, and (**C**) fresh weight of fruit of pepper plants. fresh weight of fruits per plant. Data are mean ± SD (n = 3). Different letters above the error bars indicate significant differences for *p* ≤ 0.05 (ANOVA and Tukey’s post hoc test); P = peat; SM = substrate mix; CH = chitosan; FW = fresh weight.

**Figure 4:**
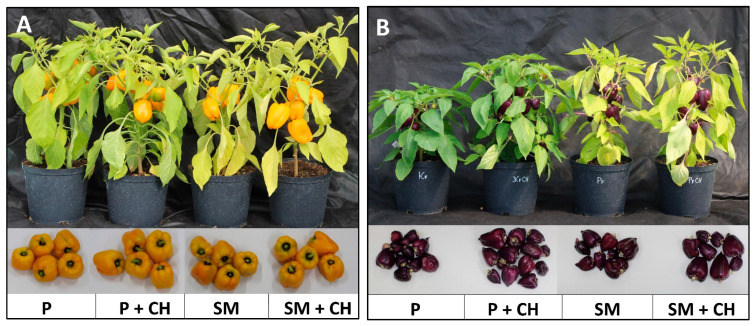
Appearance of ‘Marta Polka’ (**A**) and ‘Oda’ (**B**) pepper plants and fruits. P = peat; SM = substrate mix; CH = chitosan.

Fruit yield was affected by experimental factors and their interaction effects (Figure 3 and Figure 4). The fruit number was highest in ‘Oda’ plants grown in 100% peat and not treated with a biostimulant (14.33 per plant). The lower number of fruit was obtained from ‘Marta Polka’ cultivar grown in 100% peat and not treated with chitosan (4.33 per plant). The highest total fruit fresh weight was obtained by plants of the ‘Marta Polka’ cultivar grown in 100% peat and drenched with a depolymerized chitosan solution (471.23 g per plant), and the lowest by plants of the ‘Oda’ cultivar grown in substrate mix and not treated with a biostimulant (192.02 g per plant). The weight of a single fruit was highest in ‘Marta Polka’ cultivar grown in substrate mix and not treated with chitosan (55.44 g). The plants of the ‘Oda’ cultivar growing in the 100% peat substrate and not treated with a biostimulant had the lowest fresh weight of fruit (15.98 g). The fresh weight of ‘Oda’ fruit was significantly higher in plants grown in substrate mix with chitosan treatment (Figure 3).

The main effect of the growing medium, chitosan applied, and cultivars on the yield parameters are reported in Table 3. In general, plants grown in 100% peat produced more fruit per plant than those grown in a substrate mix. In addition, the fruit yield expressed as total fresh fruit weight per plant was also higher when the substrate was pure peat. On the other hand, the weight of a single fruit in plants grown in substrate mix was slightly higher compared to the weight of fruit harvested from plants grown in 100% peat (Table 3). Drenching the plants with a depolymerized chitosan solution reduced the total fresh fruit weight per plant, but increased the weight of a single fruit. The pepper cultivars tested differed significantly in terms of yield. The ‘Oda’ cultivar produced more fruit per plant than ‘Marta Polka’. In turn, the ‘Marta Polka’ cultivar showed significantly higher total fresh weight and single fruit weight than the ‘Oda’ cultivar (Table 3, Figure 4).

**Figure 5:**
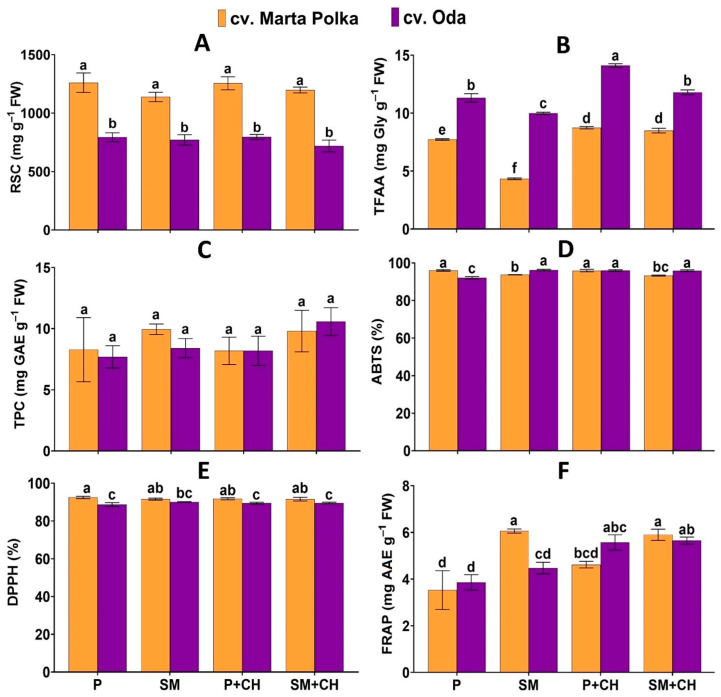
The interaction effects of media, chitosan and cultivar on (**A**) reducing sugars content (RSC); (**B**) total free amino acid content (TFAA); (**C**) total phenolic content (TPC); (**D**) antioxidant activity determined by 2,2′-Azobis(3-Ethylbenzothiazoline-6-Sulfonate) (ABTS); (**E**) antioxidant activity determined by 1,1-Diphenyl-2-Picryl-Hydrazyl (DPPH), and (**F**) antioxidant activity determined by the Ferric Reducing Antioxidant Power (FRAP) in pepper fruits. Data are mean ± SD (n = 3). Different letters above the error bars indicate significant differences for *p* ≤ 0.05 (ANOVA and Tukey’s post hoc test); P = peat; SM = substrate mix; CH = chitosan; FW = fresh weight; Gly = glycine; GAE = gallic acid equivalents;), AAE = ascorbic acid equivalent.

The effects of the treatments on the biochemical characteristics of the pepper fruits are summarized in Figure 5 and Table 4. The highest total free amino acid content were obtained in ‘Oda’ peppers grown in 100% peat drenched with a depolymerized chitosan solution (14.10 mg Gly g^−1^ FW). The highest ABTS values (96.22%) were obtained in ‘Oda’ cultivar grown in a substrate mix and not treated with a biostimulant. Extracts from the ‘Marta Polka’ cultivar exposed to 100% peat without a biostimulant had the highest antioxidant activity measured by DPPH (92.46%). The highest ABTS activity (92.46%) were obtained in ‘Oda’ cultivar grown in a substrate mix without chitosan. The highest FRAP activity (6.06 mg AAE g^−1^ FW) was observed in extracts from ‘Marta Polka’ pepper fruits harvested from plants grown in a substrate mix without biostimulant application. The highest activity of FRAP (5.65 mg AAE g^−1^ FW) was obtained for the fruits of cultivar ‘Oda’ grown in mix substrate drenched with a depolymerized chitosan. In both cultivars, the combined factors did not significantly affect the levels of reducing sugars or total polyphenols (Figure 5).

The main effects of the growing media, chitosan application and cultivar on biochemical constituents of pepper fruit reported in Table 4. Regardless of the biostimulant treatment and cultivar, the use of 100% peat resulted in a significant increase in reducing sugars and total free amino acids, and decrease total phenolic content and antioxidant activity determined by FRAP in pepper fruits compared with fruits from plants grown in substrate mix. Comparing only the effect of the biostimulant, the use of depolymerized chitosan increased total free amino acids and antioxidant activity determined by ABTS and FRAP. Results of grand mean show that pepper cultivars had similar total phenolic content in their fruits, but differed significantly in terms of reducing sugars and total free amino acids. ‘Marta Polka’ had higher reducing sugars than ‘Oda’, whereas ‘Oda’ exhibited a higher total content of free amino acids than the ‘Marta Polka’ pepper (Table 4).

## 3. Discussion

### 3.1. Contrasting Impacts of Media on Pepper

In this study, wood fiber obtained from Scots pine (*Pinus sylvestris*) chips and green compost derived from cut grass, branches, and leaves from the maintenance of green areas were used as substitutes for peat. It was shown that both pepper cultivars grown in a peat enriched with wood fiber and compost produced fruit, while their leaves contained less photosynthetic pigments and reducing sugars (Figure 1). Photographic documentation (Figure 4) confirm that the leaves of cvs. ‘Marta Polka’ and ‘Oda’ grown in a substrate mix were less green than the leaves of plants growing in 100% peat. The demonstrated reduction in chlorophyll and photoassimilate content may be related to reduced nitrogen availability in the mix substrate. Wood fiber has a high C:N ratio, and therefore, the microorganisms that decompose it require large amounts of nitrogen [50]. In our study, compost was added to the peat and fiber mixture to limit nitrogen immobilization, but no mineral fertilizer was used. In earlier studies [58], interspecific geraniums grown in a substrate containing up to 40% (*v*:*v*) wood fiber without additional nitrogen fertilization showed drastic inhibition of growth and flowering, and significantly reduced chlorophyll and nitrogen indices in the leaves. Similarly, in studies by Čepulienė et al. [59], the chlorophyll index of cucumber was reduced when plants were grown in a peat-wood fiber (50:50, *v*:*v*) mixture without additional nitrogen fertilization. In the current study, the decrease in chlorophyll and reducing sugars was accompanied by an increase in the total polyphenol content in the leaves of the ‘Marta Polka’ cultivar (Figure 2). It may indicate the involvement of this group of compounds in plant adaptation to stress conditions, most likely due to insufficient nitrogen availability in the substrate (Table 5). In general, nitrogen deficiency promotes the accumulation of total polyphenols in plants [60], including peppers [61].

‘Marta Polka’ and ‘Oda’ plants grown in a substrate with added wood fiber and compost showed reduced overall yield, measured as fruit quantity and total weight (Figure 3). It seems that one of the reasons for the lower yield in plants grown in a substrate amended with wood fiber and compost was the previously mentioned reduction in chlorophyll and soluble sugars, and possibly due to insufficient nitrogen availability. The lower water-holding capacity of wood fiber compared to peat (Table 6) may also have contributed indirectly to the decrease in pepper yield. This assumption is confirmed by the grand mean obtained for leaf stomatal conductance. Irrespectively of the biostimulant and cultivars studied, plants grown in a substrate enriched with wood fiber and compost had slightly reduced leaf stomatal conductance compared to plants grown in peat alone (Table 1). Both wood fiber and compost have lower water retention capacity than peat, which, in practice, may affect the availability of water to plants [62].

Although the overall yield of both pepper cultivars was lower when the plants were grown in a substrate enriched with wood fiber and compost, the weight of individual fruit (cv. ‘Oda’) and quality of fruits were better than those of fruits from plants grown in peat. ‘Marta Polka’ and ‘Oda’ cultivars grown in substrate mix had greater antioxidant activity determined by FRAP than the plants grown in the peat alone (Figure 5). As is well known, peppers are a rich source of antioxidants with enormous therapeutic potential [32,33,35]. It is possible that increased antioxidant properties of the extracts in plants grown in a substrate mix may have resulted from stress caused by macronutrient deficiency. Low doses of stressors can have beneficial effects on yield quality, a phenomenon known as “positive stress” or eustress [63]. Obtaining better fruit quality may also be associated with the use of compost as a substrate additive. The increase in the content of antioxidants in peppers resulting from compost use has been demonstrated in numerous studies [36,48,57]. In addition to minerals, compost contains small-molecule amino acids, vitamins, enzymes, and plant hormones that can affect the phytochemical composition and antioxidant activity of fruits [64,65,66].

### 3.2. Chitosan: From Biostimulation to Elicitation

The term ‘chitosan’ refers to a broad group of polycationic macromolecular compounds that differ mainly in molecular weight [67]. In these studies, water-soluble chitosan with a defined molecular weight of 48,000 g mol^−1^, obtained via controlled free-radical degradation, was used [68]. The application of depolymerized chitosan in the form of plant drenching altered photosynthetic parameters (Table 1). ‘Marta Polka’ and ‘Oda’ plants grown in a substrate enriched with wood fiber and compost, treatment with chitosan clearly increased net CO_2_ assimilation rate (Figure 1). These results are consistent with earlier reports in the literature indicating that chitosan can enhance gas exchange efficiency in plants [69,70,71]. On the other hand, the experiments conducted showed a decrease in chlorophyll and reducing sugars content in leaves of both pepper cultivars grown in 100% peat with chitosan (Figure 2). Such divergent effects may suggest that chitosan acted as an elicitor rather than a typical biostimulant in this case. The observed increase in photosynthetic activity may have resulted from priming, possibly involving increased activity of photosynthetic enzymes to compensate for energy losses associated with chitosan-induced defense responses [72]. Due to their structural similarity to biotic elicitors, chitosan derivatives can stimulate plant metabolism, including pathways involved in stress defense responses [73,74]. As part of this response, the plant, preparing for potential stress, increases its metabolic efficiency (including photosynthetic capacity) and strengthens its antioxidant system [75,76]. The results on the chemical composition of pepper fruits support these assumptions. Both pepper cultivars treated with chitosan showed increased antioxidant activity determined by FRAP (Figure 5), indicating the activation of defense mechanisms induced by this biopolymer. Interactions among substrate type, chitosan application, and pepper cultivar revealed complex relationships, particularly for photosynthetic and yield parameters, photosynthetic pigments and antioxidant activity indicating the importance of matching the cultivation method to the cultivar. A few studies confirm that the composition of the substrate can modify the effect of chitosan at the morphological, physiological, and metabolic levels of plants [77,78]. It has previously been reported that chitosan can affect the growth, yield, and chemical composition of peppers. Durmus et al. [79] showed that foliar spraying of ‘Sehzade F1′ peppers with chitosan with low molecular weight dissolved in acetic acid solution resulted in a decrease in the SPAD greening index, an increase in total polyphenol content, and an increase in the number of fruits and their total weight. Mawale and Giridhar [47] found that foliar treatment with chitosan (molecular weight 50,000–190,000 Da, degree of deacetylation 75%) in the form of nanoparticles on three varieties of chili peppers increased the content of chlorophyll, total polyphenols, flavonoids, and antioxidant activity (FRAP and DPPH tests) of the fruits. In turn, Asgari-Targhi et al. [80] showed that chitosan (molecular weight 110 kDa, degree of deacetylation 85–90%) added to the medium in vitro in its native form (100 mg/L) and nanoparticles (5–20 mg/L) had a phytotoxic effect and drastically inhibited the growth and development of peppers. Contradictory results indicate the need for further research on the effectiveness of chitosan as a biostimulant/elicitor.

## 4. Materials and Methods

### 4.1. Plant Material, Pot Culture Experiment, and Growing Conditions

The study evaluated two cultivars of bell pepper, ‘Marta Polka’ with yellow fruit and ‘Oda’ with purple fruit, obtained from Breeding and Seed Company W. Legutko (Jutrosin, Poland). The seeds were sown in mid-March, 2020 in black plastic sowing trays measuring 31.5 cm × 52.5 cm filled with TS1 standard substrate (Klasmann-Deilmann GmbH, Geeste, Germany). The composition of TS1 was as follows: light peat 0−5 mm, pH 5.7, PGMix NPK 14:16:18 + micro 1 g dm^−3^ fertilizer. The seed trays were placed on tables under natural photoperiod conditions in the greenhouse of the West Pomeranian University of Technology in Szczecin (53°25′ N, 14°32′ E), where the temperature during the day was 20–24 °C and 18–20 °C at night. The aligned seedlings with one pair of leaves were individually transplanted into 1 dm^3^ pots filled with TS1 substrate. The pots were placed on tables in the research greenhouse. Plants at the three-leaf stage were transplanted into 5 dm^3^ pots with two substrates: 100% peat and a substrate mix (50% peat + 25% wood fiber + 25% compost; *v*/*v*/*v*). The chemical composition and physical properties of the individual components of the substrates and substrate mix are presented in Table 6 and Table 7.

The source of nutrients was Yara Hydrocomplex fertilizer (Oslo, Norway) added to peat at a dose of 2 g dm^−3^. The plants were placed on tables in a greenhouse on 5 May 2020, and after 10 days, treatment with a biostimulant was started. The plants were drenched with a solution of degraded chitosan 8 times, every 3 days, at a concentration of 50 mg dm^−3^, using approximately 100 cm^3^ of solution per plant each time. Dose of 50 mg dm^−3^ was adopted based on previous studies [81]. The control plants were drenched with distilled water. Degraded chitosan with molecular weight ≈ 48,000 g mol^−1^ and degree of deacetylation ≈ 85%, obtained by a free radical degradation process from native chitosan (Yuhuan Ocean Biochemical Co., Ltd., Taizhou, China) at the Center for Bioimmobilization and Innovative Packaging Materials of the West Pomeranian University of Technology in Szczecin.

The experiment was set up in a three-factor system: cultivar (C) × medium (M) × biostimulant (B). A total of 8 variants were created. Each experimental variant consisted of 16 plants, with four plants per repetition (Table 7).

### 4.2. Measurements of Physiological Parameters

The gas exchange parameters, i.e., assimilation intensity of CO_2_ net (A), transpiration intensity (E), and stomatal conductivity of H_2_O, were measured using a TPS-2 portable gas analyzer with a PLC-4 (PP Systems, Amesbury, MA, USA). The photosynthetic water-use efficiency factor (ω_W_) was determined based on the A/E quotient. Measurements were taken in 12 replicates. The fluorescence parameter of chlorophyll “a”, the maximum potential efficiency of the photochemical reaction in PS II, determined after dark adaptation, following reduction of acceptors in PS II (F_V_/F_M_), was determined using a Handy PEA spectrofluorometer (Hansatech Ltd., Kings Lynn, UK), based on the standard procedure of the apparatus (3 × 650 nm LED, maximum actinic light intensity 3000 μmol m^−2^ s^−1^. The measurement was carried out for each variant on 20 randomly selected leaves in a previously darkened area for 20 min, using factory-made clips (irradiation area: 4 mm). The measurements were performed on healthy, undamaged leaves in the middle part of the plants. In the ‘Oda’ cultivar, the leaves with petioles were on average 20 cm long, with a leaf blade 15 cm long and 7.2 cm wide. In the ‘Marta Polka’ cultivar, the leaves with petioles were on average 21.5 cm long, with a leaf blade 16 cm long and 7.2 cm wide.

### 4.3. Measurements of Plant Yield

The fruit yield was assessed once in the ‘Oda’ cultivar on 4 August 2020, and in the ‘Marta Polka’ cultivar on 4 September 2020. All ripe fruits were harvested separately from each plant, counted, and weighed. The number of fruits per plant, the total fresh weight of fruits per plant, and the fresh weight of a single fruit were determined.

### 4.4. Biochemical Analyses

#### 4.4.1. Preparation of Leaves and Fruits for Analysis

For the analyses, five leaves (average fresh leaf weight 19.4 g per plant) and four fruits (average fresh fruit weight 183 g per plant) were used from each plant, which were crushed and then frozen at −20 °C for 12 h. The leaf and fruit samples were then freeze-dried for 24 h in a Beta 2–8 LSC freeze dryer plus (Martin Christ Gefriertrocknungsanlagen GmbH, Osterode am Harz, Germany) and ground into powder.

#### 4.4.2. Determination of Photosynthetic Pigment Content in Leaves

0.5 g of leaf samples were transferred to a Falcon tube and mixed with 50 mL of H_2_O-80% acetone (2:8 *v*/*v*). The samples were extracted for 5 min in an ultrasonic bath. The samples were then centrifuged at 6000× *g* for 10 min. The content of photosynthetic pigments (total chlorophyll and total carotenoids) was determined spectrophotometrically using the modified method of Grzeszczuk et al. [82]. Each sample was measured in three replicates. The total chlorophyll content was expressed in mg g^−1^ FW, and the total carotenoid content in mg 100 g^−1^ FW.

#### 4.4.3. Determination of the Total Reducing Sugars Content in Leaves and Fruits

The total reducing sugars content was determined according to the method described by Łopusiewicz et al. [83]. One milliliter of supernatant was combined with 1 mL of 0.05 M acetic acid buffer (pH 4.8) and 3 mL of 3,5-dinitrosalicylic acid (DNS) reagent. The entire mixture was shaken vigorously and then incubated for 5 min in hot water (~96 °C). The tubes were cooled to room temperature. Absorbance was measured at λ = 540 nm. Glucose in acetate buffer was used to prepare the standard curve.

#### 4.4.4. Determination of the Total Free Amino Acid Content in Leaves and Fruits

The total level of free amino acids was analyzed spectrophotometrically according to the method described by Łopusiewicz et al. [83]. One milliliter of supernatant was combined with two milliliters of ninhydrin-Cd reagent in test tubes. The samples were vortexed and heated at 84 °C (5 min), then cooled on ice. The supernatant was transferred to a plate, and the absorbance was measured at λ = 507 nm. The results were calculated as mg of glycine (Gly) equivalent per g^−1^ FW.

#### 4.4.5. Determination of Total Polyphenols in Leaves and Fruits

The total polyphenol content in the extracts was determined spectrophotometrically using a microplate reader (Synergy LX, BioTek, Winooski, VT, USA) with the Folin–Ciocalteu method described by Tong et al. [84] and our own modifications. The method involves mixing 20 µL of supernatant with 150 µL of distilled water and 100 µL of Folin–Ciocalteu reagent. The solution was supplemented with 80 µL of saturated Na_2_CO_3_ (after 5 min). Finally, the mixture was incubated in the dark at 40 °C (30 min). The absorbance was measured at 765 nm, and the polyphenol concentration was expressed as milligrams of gallic acid equivalents (GAE) per g^−1^ FW.

#### 4.4.6. 1,1-Diphenyl-2-picryl-hydrazyl (DPPH) and 2,2′-Azobis(3-ethylbenzothiazoline-6-sulfonate) (ABTS) Antioxidant Capacity Tests of Fruit

The DPPH and ABTS antioxidant capacity tests were measured according to the method described by Łopusiewicz et al. [83]. The DPPH method uses the stable DPPH˙ radical, i.e., 2,2-diphenyl-1-picrylhydrazyl. 0.5 mL of a DPPH solution in methanol (0.01 mM) was mixed with 0.5 mL of the extract in a 1:1 ratio. The samples were incubated at 22 ± 2 °C for 30 min, without exposure to light. Finally, the absorbance was measured at λ = 517 nm. The total antioxidant potential of the analyzed extracts was determined spectrophotometrically using the ABTS assay. This method involves the direct generation of ABTS^+˙^ radicals via the oxidation of ABTS by potassium persulfate. The addition of an antioxidant reduces the cation radical to ABTS and decreases the solution’s color intensity. In 1.5 mL Eppendorf tubes, 1.5 mL of ABTS solution was mixed with 25 µL of extract, and the mixture was incubated at room temperature in the dark for 10 min. Before proceeding with the measurements, the ABTS solution was diluted with a 96% ethanol solution to obtain an absorbance of 0.700 ± 0.02. Finally, the absorbance was measured at λ = 734 nm.

#### 4.4.7. Determination of the Ferric Reducing Antioxidant Power (FRAP) of Fruit

The method for determining the reducing capacity of Fe (III) ions (FRAP) is based on spectrophotometric measurement of the reduction of TPTZ (iron-2,4,6-tripyridyl-S-thiazine complex) by an antioxidant, as described by Łopusiewicz et al. [83]. The FRAP reagent was prepared by mixing 2.5 mL of TPTZ in 40 mM HCl, 2.5 mL of FeCl_3_, and 25 mL of acetate buffer (pH 3.6, 300 mM). Then, 20 µL of supernatant was transferred to a 96-well TC plate in three replicates by adding 280 µL of FRAP reagent, and the plate was gently shaken for 10 s. The absorbance was read at 595 nm. The results were expressed as ascorbic acid equivalent (AAE) per g^−1^ FW using a standard curve for ascorbic acid.

### 4.5. Statistical Analysis

The results were statistically analyzed using analysis of variance (ANOVA) for three-factor experiments in TIBCO Statistica™ Professional 13.3.0 (TIBCO Statistica, Palo Alto, CA, USA). After rejecting the null hypothesis in the analysis of variance, to determine differences between the means, a multiple-comparison test was performed using Tukey’s test, based on the homogeneity of variance previously assessed using Levene’s test.

## 5. Conclusions

In summary, we demonstrated that peat substrates amended with wood fiber and green compost have strong potential as sustainable media for container production of bell pepper in greenhouses. The ‘Oda’ cultivar responds most favorably to depolymerized chitosan in the substrate mix, showing increased net CO_2_ assimilation rate, stomatal conductance, fresh fruit weight, and antioxidant activity. The ‘Marta Polka’ plants grown in the substrate mix with chitosan exhibit improved net CO_2_ assimilation, photosynthetic water-use efficiency, free amino acid content, and antioxidant activity compared with plants grown in untreated peat. The significant interactions among substrate type, chitosan application, and cultivar further indicate that the effectiveness of biostimulation and the metabolic responses of bell peppers are strongly determined by genotype–environment interactions, which should be considered when optimizing cultivation practices in environmentally friendly production systems.

## Figures and Tables

**Table 1:** Main effects of medium, biostimulant and cultivar on assimilation intensity CO_2_ net (A), transpiration intensity (E), stomatal conductivity H_2_O (g_s_), photosynthetic water utilization factor (ω_W_), and maximum photochemical efficiency (F_V_/F_M_) of pepper plants. The data are presented as grand mean.

Treatments	A (μmol m^−2^ s^−1^)	E (mmol m^−2^ s^−1^)	ω_W_ (mmol mol^−1^)	g_S_ (mol m^−2^ s^−1^)	F_V_/F_M_
Medium (M)					
Peat	4.11 ^b^	0.92 ^b^	5.01 ^a^	0.10 ^a^	0.72 ^b^
Substrate mix	5.38 ^a^	1.08 ^a^	5.18 ^a^	0.09 ^b^	0.75 ^a^
Biostimulant (B)					
Control	3.66 ^b^	0.90 ^b^	4.69 ^b^	0.09 ^a^	0.73 ^a^
Chitosan	5.83 ^a^	1.10 ^a^	5.50 ^a^	0.10 ^a^	0.74 ^a^
Cultivar (C)					
Marta Polka	4.79 ^a^	1.06 ^a^	4.83 ^b^	0.11 ^a^	0.75 ^a^
Oda	4.69 ^a^	0.94 ^b^	5.36 ^a^	0.09 ^b^	0.72 ^a^
*p* (ANOVA) for Main Factor					
M	***	***	ns	*	*
B	***	***	**	ns	ns
C	ns	***	*	***	ns
*p* (ANOVA) for Interaction					
M × B	***	***	***	***	ns
M × C	**	ns	***	*	ns
B × C	ns	***	***	***	ns
M × B × C	ns	*	ns	**	ns

Different letters within each column indicate significant differences for *p* ≤ 0.05 (ANOVA and Tukey’s post hoc test); ns, ***, **, *, Nonsignificant or significant at *p* ≤ 0.001, 0.01 or 0.05, respectively.

**Table 2:** Main effects of medium, biostimulant and cultivar on total chlorophyll (Chl), total carotenoids (Car), reducing sugars (RSC), total free amino acid (TFAA), and total phenolic content (TPC) in the leaves of pepper plants. The data are presented as grand mean.

Treatments	Chl (mg g^−1^ FW)	Car (mg 100 g^−1^ FW)	RSC (mg g^−1^ FW)	TFAA (mg Gly g^−1^ FW)	TPC (mg GAE g^−1^ FW)
Medium (M)					
Peat	3.77 ^a^	80.94 ^a^	249.06 ^a^	3.64 ^a^	15.64 ^b^
Substrate mix	1.76 ^b^	59.63 ^b^	160.37 ^b^	2.80 ^b^	17.31 ^a^
Biostimulant (B)					
Control	2.94 ^a^	71.00 ^a^	205.61 ^a^	3.23 ^a^	16.33 ^a^
Chitosan	2.60 ^b^	69.57 ^a^	203.82 ^a^	3.21 ^a^	16.62 ^a^
Cultivar (C)					
Marta Polka	2.31 ^b^	66.17 ^b^	222.84 ^a^	3.71 ^a^	18.96 ^a^
Oda	3.23 ^a^	74.39 ^a^	186.59 ^b^	2.74 ^b^	14.00 ^b^
*p* (ANOVA) for Main Factor					
M	***	***	***	***	**
B	***	ns	ns	ns	ns
C	***	***	***	***	***
*p* (ANOVA) for Interaction					
M × B	ns	**	ns	ns	ns
M × C	**	***	***	***	*
B × C	*	ns	***	ns	ns
M × B × C	**	*	**	***	ns

Different letters within each column indicate significant differences for *p* ≤ 0.05 (ANOVA and Tukey’s post hoc test); ns, ***, **, *, Nonsignificant or significant at *p* ≤ 0.001, 0.01 or 0.05, respectively; FW = fresh weight; Gly = glycine; GAE = gallic acid equivalents.

**Table 3:** Main effects of medium, biostimulant and cultivar on total fruit number per plant, total fresh weight of fruits per plant, and fresh weight of fruit of pepper plants. The data are presented as grand mean.

Treatments	Total Fruit (no.)	Total Fruit FW (g)	FW of Fruit (g)
Medium (M)			
Peat	11.13 ^a^	360.45 ^a^	37.11 ^b^
Substrate mix	6.08 ^b^	218.68 ^b^	40.63 ^a^
Biostimulant (B)			
Control	8.63 ^a^	268.38 ^b^	37.63 ^b^
Chitosan	8.58 ^a^	310.74 ^a^	40.11 ^a^
Cultivar (C)			
Marta Polka	6.58 ^b^	344.73 ^a^	53.74 ^a^
Oda	10.63 ^a^	234.40 ^b^	23.99 ^b^
*p* (ANOVA) for Main Factor			
M	***	***	***
B	ns	***	**
C	***	***	***
*p* (ANOVA) for Interaction			
M × B	ns	**	**
M × C	***	***	***
B × C	**	ns	***
M × B × C	ns	*	ns

Different letters within each column indicate significant differences for *p* ≤ 0.05 (ANOVA and Tukey’s post hoc test); ns, ***, **, *, Nonsignificant or significant at *p* ≤ 0.001, 0.01 or 0.05, respectively; FW = fresh weight.

**Table 4:** Main effects of medium, biostimulant and cultivar on reducing sugars content (RSC), total free amino acid content (TFAA), total phenolic content (TPC) and antioxidant activity (ABTS, DPPH, FRAP) in pepper fruits. The data are presented as grand mean.

Treatments	RSC (mg g^−1^ FW)	TFAA (mg Gly g^−1^ FW)	TPC (mg GAE g^−1^ FW)	ABTS (%)	DPPH (%)	FRAP (mg AAE g^−1^ FW)
Medium (M)						
Peat	1026.50 ^a^	10.47 ^a^	8.08 ^b^	95.01 ^a^	90.59 ^a^	4.40 ^b^
Substrate mix	955.82 ^b^	8.65 ^b^	9.68 ^a^	94.78 ^a^	90.70 ^a^	5.52 ^a^
Biostimulant (B)						
Control	991.28 ^a^	8.34 ^b^	8.58 ^a^	94.52 ^b^	90.70 ^a^	4.48 ^b^
Chitosan	991.04 ^a^	10.78 ^a^	9.19 ^a^	95.27 ^a^	90.59 ^a^	5.43 ^a^
Cultivar (C)						
Marta Polka	1211.94 ^a^	7.32 ^b^	9.05 ^a^	94.73 ^a^	91.84 ^a^	5.03 ^a^
Oda	770.38 ^b^	11.80 ^a^	8.71 ^a^	95.07 ^a^	89.45 ^b^	4.89 ^a^
*p* (ANOVA) for Main Factor						
M	**	***	*	ns	ns	***
B	ns	***	ns	**	ns	***
C	***	***	ns	ns	***	ns
*p* (ANOVA) for Interaction						
M × B	ns	***	ns	***	ns	*
M × C	ns	ns	ns	***	*	**
B × C	ns	ns	ns	***	ns	**
M × B × C	ns	***	ns	***	ns	ns

Different letters within each column indicate significant differences for *p* ≤ 0.05 (ANOVA and Tukey’s post hoc test); ns, ***, **, *, Nonsignificant or significant at *p* ≤ 0.001, 0.01 or 0.05, respectively; FW = fresh weight; Gly = glycine; GAE = gallic acid equivalents; ABTS = 2,2′-Azobis(3-Ethylbenzothiazoline-6-Sulfonate), DPPH = 1,1-Diphenyl-2-Picryl-Hydrazyl, FRAP = the Ferric Reducing Antioxidant Power; AAE = ascorbic acid equivalent.

**Table 5:** Media chemical properties.

Parameters	Peat	Wood Fiber	Compost	Substrate Mix
pH (H_2_O, 1:2, *v*:*v*)	6.00	4.40	7.30	6.60
Salinity (g NaCl dm^−3^)	0.22	0.34	8.07	2.04
NO_3_–N (mg dm^−3^)	12.0	35.0	858	91
P (mg dm^−3^)	20.0	36.0	363	194
K (mg dm^−3^)	26.0	204	4570	1173
Mg (mg dm^−3^)	120	84.0	554	278
Ca (mg dm^−3^)	586	229	915	620
S (mg dm^−3^)	18.2	7.60	193	119
Fe (mg dm^−3^)	46.3	59.0	79.5	71.9
Mn (mg dm^−3^)	2.44	66.4	11.8	20.6
Cu (mg dm^−3^)	0.45	0.59	2.08	1.64
Zn (mg dm^−3^)	2.88	8.39	29.0	19.0
B (mg dm^−3^)	0.84	0.83	6.70	3.13

**Table 6:** Media physical properties.

Parameters	Peat	Wood Fiber	Compost	Substrate Mix
Moisture (%)	43.3	32.5	43.2	41.1
Shrinkage (%)	26.5	11.5	24.1	20.6
Bulk density (g cm^−3^)	0.14	0.17	0.27	0.26
Total pore space (%)	90.7	89.5	87.1	85.1
Water filled pore space (%)	63.4	39.4	44.2	45.7
Air filled pore space (%)	27.3	50.1	42.9	39.4

**Table 7:** Treatments and experimental setup.

Treatment	Cultivar	Medium	Biostimulant	No. of Plants	Replicates
1	Marta Polka	100% Peat	Control	16	4 × 4
2	Marta Polka	100% Peat	Chitosan	16	4 × 4
3	Marta Polka	Substrate mix	Control	16	4 × 4
4	Marta Polka	Substrate mix	Chitosan	16	4 × 4
5	Oda	100% Peat	Control	16	4 × 4
6	Oda	100% Peat	Chitosan	16	4 × 4
7	Oda	Substrate mix	Control	16	4 × 4
8	Oda	Substrate mix	Chitosan	16	4 × 4

## Data Availability

The data presented in this study are available on request from the corresponding author.
